# Supplementary material for: Resolving the full spectrum of human genome variation using Linked-Reads
Source: Genome Res. 2019 Apr;29(4):635–45. doi: 10.1101/gr.234443.118 (PMC6442396; doi:10.1101/gr.234443.118)
Supplement: Supplemental Material [file supp_gr.234443.118_Supplemental_longranger-2.2.zip › longranger-2.2.tar.bz2!/lr2.2-public/lib/stan/cmdstan/stan/doc/api/html/agrad_2rev_2functions_2log1m__exp_8hpp_source.html]

Stan: src/stan/agrad/rev/functions/log1m\_exp.hpp Source File


|  |  |
| --- | --- |
|  | Stan  2.5.0  probability, sampling & optimization |


- Main Page
- Related Pages
- Namespaces
- Classes
- Files

- File List
- File Members

All Classes Namespaces Files Functions Variables Typedefs Enumerations Enumerator Friends Macros Pages

- src
- stan
- agrad
- rev
- functions

log1m\_exp.hpp

Go to the documentation of this file.

1 #ifndef STAN\_\_AGRAD\_\_REV\_\_FUNCTIONS\_\_LOG1M\_EXP\_HPP

2 #define STAN\_\_AGRAD\_\_REV\_\_FUNCTIONS\_\_LOG1M\_EXP\_HPP

3

4 #include <stan/agrad/rev/var.hpp>

5 #include <stan/agrad/rev/internal/v\_vari.hpp>

6 #include <stan/math/functions/log1m\_exp.hpp>

7 #include <stan/agrad/rev/calculate\_chain.hpp>

8 #include <boost/math/special\_functions/expm1.hpp>

9

10 namespace stan {

11  namespace agrad {

12

13  namespace {

14  class log1m\_exp\_v\_vari : public op\_v\_vari {

15  public:

16  log1m\_exp\_v\_vari(vari\* avi) :

17  op\_v\_vari(stan::math::log1m\_exp(avi->val\_),

18  avi) {

19  }

20  void chain() {

21  //derivative of log(1-exp(x)) = -exp(x)/(1-exp(x)) = -1/(exp(-x)-1) = -1/expm1(-x)

22  avi\_->adj\_ -= adj\_ / boost::math::expm1(-(avi\_->val\_));

23  }

24  };

25  }

26

31  inline var log1m\_exp(const stan::agrad::var& a) {

32  return var(new log1m\_exp\_v\_vari(a.vi\_));

33  }

34

35  }

36 }

37 #endif

log1m\_exp.hpp

stan::agrad::var::vi\_

vari \* vi\_

Pointer to the implementation of this variable.

**Definition:** var.hpp:40

stan::agrad::expm1

fvar< T > expm1(const fvar< T > &x)

**Definition:** expm1.hpp:15

v\_vari.hpp

var.hpp

stan::agrad::log1m\_exp

fvar< T > log1m\_exp(const fvar< T > &x)

**Definition:** log1m\_exp.hpp:17

stan::agrad::var

Independent (input) and dependent (output) variables for gradients.

**Definition:** var.hpp:27

calculate\_chain.hpp


---

[ Stan Home Page ]

*© 2011–2014,
Stan Development Team.*
